# Vaccination against δ−Retroviruses: The Bovine Leukemia Virus Paradigm

**DOI:** 10.3390/v6062416

**Published:** 2014-06-20

**Authors:** Gerónimo Gutiérrez, Sabrina M. Rodríguez, Alix de Brogniez, Nicolas Gillet, Ramarao Golime, Arsène Burny, Juan-Pablo Jaworski, Irene Alvarez, Lucas Vagnoni, Karina Trono, Luc Willems

**Affiliations:** 1 Instituto de Virología, Centro de Investigaciones en Ciencias Veterinarias y Agronómicas, INTA, C.C. 1712, Castelar, Argentina; E-Mails: gutierrez.geronimo@inta.gob.ar (G.G.); jaworski.juan@inta.gob.ar (J.-P.J.); alvarez.irene@inta.gob.ar (I.A.); vagnoni.lucas@inta.gob.ar (L.V.); trono.karina@inta.gob.ar (K.T.); 2 Molecular and Cellular Epigenetics (GIGA) and Molecular Biology (Gembloux Agro-Bio Tech), University of Liège (ULg), 4000 Liège, Belgium; E-Mails: sabrirodriguez@hotmail.com (S.M.R.); Alix.deBrogniez@student.ulg.ac.be (D.B.); n.gillet@ulg.ac.be (N.G.); ramugolime@gmail.com (R.G.); arsene.burny@guest.ulg.ac.be (A.B.)

**Keywords:** BLV, neutralizing antibody, vaccine, mimotope, HTLV-1, attenuated virus

## Abstract

Bovine leukemia virus (BLV) and human T-lymphotropic virus type 1 (HTLV-1) are closely related δ-retroviruses that induce hematological diseases. HTLV-1 infects about 15 million people worldwide, mainly in subtropical areas. HTLV-1 induces a wide spectrum of diseases (e.g., HTLV-associated myelopathy/tropical spastic paraparesis) and leukemia/lymphoma (adult T-cell leukemia). Bovine leukemia virus is a major pathogen of cattle, causing important economic losses due to a reduction in production, export limitations and lymphoma-associated death. In the absence of satisfactory treatment for these diseases and besides the prevention of transmission, the best option to reduce the prevalence of δ-retroviruses is vaccination. Here, we provide an overview of the different vaccination strategies in the BLV model and outline key parameters required for vaccine efficacy.

## 1. Clinical Course

**Figure 1:**
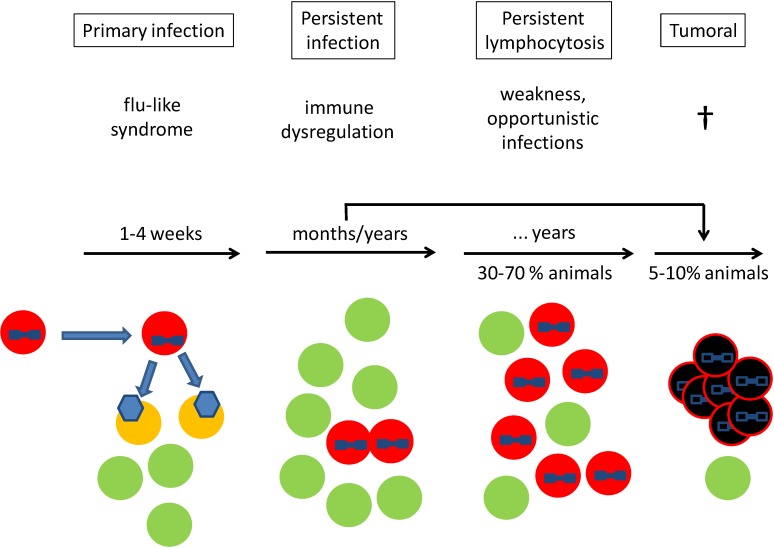
Clinical course of bovine leukemia virus (BLV) infection. Primary infection: an infected cell (red) with a BLV integrated into the host chromosome (blue provirus) is transmitted into a new animal. During primary infection, the BLV provirus is expressed into viral particles (blue hexagon), which further infect B-cells (yellow). Active BLV replication is responsible for a “flu-like” syndrome, as observed during primary infection by HIV in humans. During persistent infection, provirus-carrying cells (red) expand mainly by mitosis, because of the presence of an active immune response. This phase extends for several months/years and is characterized by an immune dysregulation (e.g., overexpression of cytokines), as observed in HTLV associated myelopathy / tropical spastic paraparesis (HAM/TSP) subjects infected by human T-lymphotropic virus type 1 (HTLV-1). In about 30%–70% of animals, the number of infected cells in blood increases above normal levels of 10,000/mm^3^. During this persistent lymphocytosis phase, morbidity is characterized by weakness and opportunistic infections, as observed in chronic lymphocytic leukemia in human. Morbidity (e.g., mastitis) leads to a loss in milk production. In the tumor phase, a single infected cell undergoes genetic mutations (black) and forms a lymphoma within or outside lymph nodes, leading to the death of the animal. Typically, animals undergo sudden death from the hemorrhage of the spleen. Tumors can also occur directly in persistently infected animals without persistent lymphocytosis (PL). The frequency of tumors and clinical latency depend on herd prevalence. A typical picture is 10% death after three years in a herd having 50% BLV prevalence.

Bovine leukemia virus (BLV) is a δ-retrovirus that infects B-lymphocytes and induces a persistent infection in cattle with diverse clinical outcomes [1,2,3]. Viral transmission occurs through the transfer of BLV-positive cells present in blood or milk from an infected animal to a new host (Figure 1). Then, the BLV virus actively replicates and infects a population of new target cells (*i.e.*, the replicative cycle). After a few weeks, a very efficient immune response strongly limits the infection of new target cells [4]. The infected lymphocytes will then proliferate and expand (*i.e.*, the clonal expansion or mitotic cycle). In fact, the majority of BLV-infected animals (around 70%) are mostly asymptomatic carriers of the virus. In these animals, neither clinical symptoms nor the alteration of the total lymphocyte counts are clearly evidenced.

After a latency that extends from a few months to several years, 30%–50% of BLV-infected animals develop a polyclonal proliferation of B-cells, called persistent lymphocytosis (PL). Polyclonal expansion means that different B-cell clones carrying a BLV virus integrated in the genome proliferate during PL (Figure 1). This clinical condition is characterized by an increase in the absolute number of peripheral blood circulating B-lymphocytes (above 10,000/mm^3^). B-lymphocytes also become more abundant than T-lymphocytes, causing an inversion of the B/T ratio. PL itself is a subclinical feature, but these animals may suffer from disturbances of the immune system, as evidenced by opportunistic infections (e.g., mastitis). In this immune dysregulation, upregulation of inhibitory receptor molecules in T-cells, induced by BLV, may play a role in the progression of the disease and susceptibility to other infections [4,5]. PL is usually stable for several years, but can also progress to the tumor phase. Because the probability of tumor development is greater in animals harboring increased levels of circulating lymphocytes, PL in cattle can be considered as a pre-tumoral stage [1].

The most conspicuous clinical manifestation of BLV infection is the development of tumors in lymphoid organs (lymph nodes, spleen), as well as in other tissues. This condition, called lymphoma, occurs in about 5%–10% of infected animals, predominantly in adult cattle older than 3–5 years (Figure 1). Unlike PL, the expansion of infected cells is of mono- or oligo-clonal origin, meaning that only a single or a few infected cells generate the tumor after multiple divisions. Besides an impact on survival, BLV-induced tumors also disturb the immune system, leading to opportunistic infections, as observed in PL.

## 2. Molecular Mechanisms of Infection and Pathogenesis

**Figure 2:**
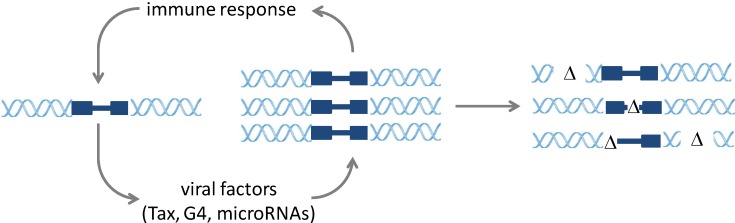
The onset of mutations during BLV infection. During infection, BLV-encoded oncogenes (Tax, G4 and, perhaps, microRNAs) stimulate proviral replication. This clonal expansion faces tight control by the host immune response. Successive replication/destruction cycles lead to the onset of replication errors in the viral and host cell genomes (Δ).

In the absence of major clinical manifestations, chronic BLV infection is, however, not latent, as evidenced by the permanent stimulation of the host immune response. Cell kinetic experiments based on intravenous injection of bromodeoxyuridine (BrdU) and carboxyfluorescein succinimidyl ester (CFSE) clearly demonstrated that infected cells undergo active replication [6,7,8]. In fact, the replication rate of infected cells should slightly exceed the normal level to persist and expand in the host [9]. Once integrated in the cell genome, the BLV provirus undergoes successive rounds of active transcription and gene silencing. Although the precise mechanisms need further characterization, it is likely that the initial signal leading to viral expression is stimulation through the B-cell receptor [10,11]. Once initiated, viral expression is activated through the Tax protein that interacts with the 5' LTR promoter through the CREB cellular factor [12,13]. Viral expression is further regulated post-transcriptionally by other proteins, such as Rex and R3 [14,15]. Upon translation of all other structural proteins, a viral particle is assembled, incorporating the genomic RNA in the newly made capsid and budding at the cell membrane. The half-life of this cell presenting viral antigen is, however, very short, due to the presence of a very active immune response during the whole infection process. For this reason, viremia (*i.e.*, the presence of viral particles in the blood plasma) is absent in infected animals, only very rare cells with viral transcripts being identified *in vivo* [16]. This permanent stimulation by viral antigens boosts a very active cytotoxic and humoral response. Besides the production of new virions, virally-encoded proteins (such as Tax and G4) and, perhaps, microRNAs concomitantly stimulate cell proliferation and duplication of the proviral clone [17,18,19,20]. Provided that the cell escapes the immune response upon the silencing of viral expression, the integrated provirus can replicate by mitosis of its host cell (Figure 2).

Although their role is still unknown, the recent discovery of microRNAs added an additional level of complexity, but also provided a possible alternate mode of clonal expansion independent of viral proteins. We infer, however, that these microRNAs are largely dispensable for infection and have weak effects on viral replication once infection is established [21]. The chronic phase of infection is thus characterized by successive cycles of virus-stimulated cell replications. Different cell clones expand or shrink as a consequence of either oncogene-driven proliferation or destruction by the host immune response. This permanent oncogenic stress engineered by viral factors is believed to trigger DNA instability, materialized by genomic mutations or deletions [22,23]. Some of these mutations provide a selective advantage to the clone and act as drivers of infected cell expansion. Ultimately, these genomic alterations also lead to tumor development. At the final stages, the provirus itself undergoes mutations and deletions, leading to the replication of defective clones that nevertheless still express microRNAs.

Although still speculative, this model recapitulates the current knowledge and elucidates some of the mechanisms involved.

## 3. Preventive Strategies

Since the virion is very unstable, BLV is likely transferred via an infected cell: a B-lymphocyte during iatrogenic procedures (dehorning, tattooing, needle sharing) and milk cells (macrophages and lymphocytes) in cow-to-calf transmission [24]. About 6% of infections occur *in utero* by unknown mechanisms that could involve the intermittent infection of antigen presenting cells or trans-cell migration, as described for human T-lymphotropic virus type 1 (HTLV-1) [25]. Although still controversial, transmission via blood suckling insects, such as tabanids, may be possible in particular conditions [26]. Management strategies have been designed and tested to counter/target these different routes of infection. Practices include: (i) the use of disposable materials (e.g., needles, syringes, obstetrical sleeves); (ii) sterilization of instruments used in procedures, such as dehorning, tattooing, castration or ear-tagging; (iii) disinfection of milking machines; (iv) equipment implementation (e.g., electrical or gas burning devices rather than gouging equipment during dehorning); (v) the use of pasteurized colostrum from BLV-infected cows or milk replacer; (vi) the elimination of insects; and (vii) artificial insemination and embryo transfer with BLV-free dams and bulls. Other implementing measures could be quarantine, the reduction of movement and isolation of calves in individual hutches. Preventive measures have been efficient to reduce the clinical impact of BLV infection, but did not achieve complete clearance of the virus. In fact, the efficacy of this strategy based exclusively on strict sanitary measures is still controversial.

An alternative approach is the identification of infected animals by hematologic (leucocyte counts), genomic (PCR) or serologic (ELISA) methods and the removal of infected animals from the herd and/or slaughtering. Evidence of the efficiency of this strategy is illustrated by the successful eradication of the disease in several countries of Western Europe. Besides the loss of genetic and reproductive potential, the cost can quickly become prohibitive in regions with intermediate prevalence. Under these conditions, an approach based on the segregation of animals with high proviral loads would be more appropriate, as high proviral load animals should be considered as having a higher probability of transmission of infection. Although potentially efficient, this approach is, however, too expensive in regions with high BLV prevalence [27].

Finally, it should be mentioned that, although host genetic factors of susceptibility to infection have been identified in the bovine genome, successful selection of BLV-resistant cattle has not been reported.

Together, these failures and limitations reveal that an effective vaccine may be the most efficient and cost-effective mode of protection.

## 4. Previous Failures in Vaccine Development against BLV

Development of a vaccine against BLV is facilitated by the relatively high stability of its genome contrasting with the quasispecies divergence in HIV [28]. Another important feature is that antibodies from the maternal colostrum protect from BLV infection [29]. However, the titers of these neutralizing antibodies rapidly decrease, leaving an open door for subsequent infections.

During the last few decades, a broad series of attempts were performed to develop a vaccine against BLV (reviewed in [24] and the references therein). Early studies evaluated preparations of inactivated BLV or crude lysates from persistently infected cell lines. Although some of these trials led to partial protection, this strategy has the intrinsic risk of transmitting infection. Therefore, viral proteins, such as gp51 surface envelope glycoprotein or p24 gag antigen, were tested for prophylactic immunization. Although some of these vaccine preparations were immunogenic, they did not consistently protect from BLV challenge [30,31]. Similar conclusions were obtained with short peptides, possibly due to inadequate stereochemical structure and partial epitope presentation [32,33,34]. In fact, the main limitations of these vaccines include the fast decline of protective antibody titers and poor stimulation of cytotoxic response. Therefore, these vaccination strategies were not successful when evaluated in herd conditions.

Another major strategy used recombinant vaccinia virus (RVV) as a vehicle for immunization against BLV antigens. Arguments in favor of this approach included a broad host range specificity, the capacity to carry a large amount of genetic information and, most importantly, the ability to elicit both humoral and cell-mediated immunity. Although RVV expressing BLV envelope glycoproteins only conferred partial protection, the proviral loads were nevertheless reduced in vaccinated sheep used as an experimental model, suggesting a potential use in therapeutic development [35,36]. For still unclear reasons, the RVV-env vaccines were unfortunately ineffective in cows.

Another series of trials included DNA vaccines, such as vectors containing the *env* and *tax* genes under the control of the cytomegalovirus or Srα promoters [37,38,39]. Although these DNA vaccines elicited a vigorous immune response, they did not prevent later infection, most likely due to transient activation. As other previously developed immunogens, DNA vaccines were thus also disappointing.

## 5. Competitive Attenuated Proviruses

Previous trials thus indicated that the failure of traditional vaccines was likely due to inadequate or short-lived stimulation of all immunity components. Ideally, the optimal vaccine would therefore contain a large number of viral factors permanently stimulating the immune response. Attenuated derivatives of BLV proviruses met these requirements. A first generation of recombinants containing spleen necrosis virus (SNV) regulatory sequences and BLV proviruses lacking tax, rex, R3 and G4 genes was designed [40]. These hybrid viruses were infectious and induced specific antibody responses in rat and rabbit models [41]. The efficacy of this vaccine in cows is unknown, but faces the restrictions of recombinant DNA technology and potential hazards of hybrid viruses.

**Figure 3:**
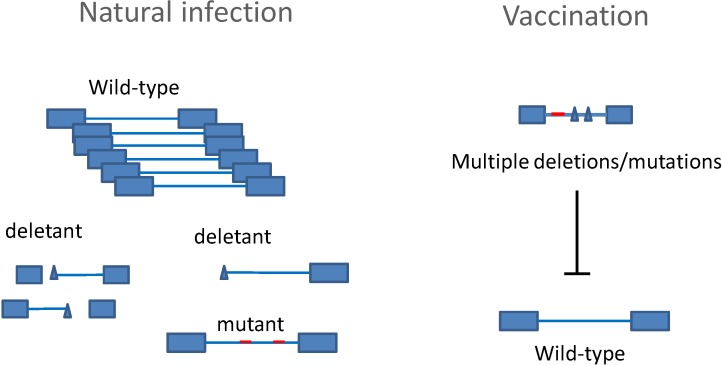
Vaccination strategy using an attenuated BLV strain. During chronic infection, mutations and deletions naturally occur in the BLV provirus, leading either to viruses with defective replication or to attenuated strains. This type of deletant or mutant provirus can be used as a vaccine, provided that it lacks pathogenicity and boosts all components of the anti-viral response.

Another approach to design attenuated BLV viruses was to delete genes dispensable for infectivity, but required for efficient replication [24]. The concept of this approach is to establish a permanent infection with an attenuated strain that impedes wild-type challenge by activating the anti-viral immune response. In fact, deletions and mutations naturally occur in BLV proviruses, frequently leading to replication-defective or attenuated clones. The idea was to design and inoculate this type of attenuated strain prior to wild-type infection (Figure 3). Using an infectious molecular clone, a series of BLV mutant or deletant proviruses were engineered and evaluated for infectivity, replication and pathogenicity in sheep. The challenge was to obtain a strain that was sufficiently impaired for pathogenicity, but that was still able to induce a persistent and broad antiviral response. This type of virus with multiple deletions and mutations was finally obtained after about two decades of test-trial cycles [42]. The vaccine strain contained deletions of potentially harmful sequences (Tax, G4, microRNAs) combined with other mutations in genes required for infectivity and replication (TM and R3). The most critical parameter was to control proviral loads that correlate closely with the risk of leukemia and the transmission of infection. Moreover, a low level of replication would also prevent a significant impact on the host immunocompetence, thereby avoiding immunosuppression and opportunistic infections.

Vaccinated animals could be dissociated from wild-type infection using PCR and possibly via immunological techniques, such as ELISA. The vaccine was infectious and replicated at low levels, but elicited a vigorous immune response in 13 animals. This attenuated strain conferred long-term protection after the challenge of cattle with a high dose of wild-type virus. The safety of the vaccine was supported by the lack of infection of control sentinels over a five-year period in herd conditions. Although the vaccine was not transmitted from cow-to-calf in 12 animals, maternal milk provided anti-BLV passive immunization. Trials are currently ongoing to evaluate the efficacy and safety of this competitive infection strategy in large herds.

## 6. A BLV Vaccine as Vector Producing HTLV Neutralizing Antibodies

An attenuated vaccine thus appears to be the only efficient method that protects from BLV infection, mainly because the different components of the immune response need to be permanently stimulated. In HTLV, it is not conceivable to use this type of strategy in non-infected subjects, because of an unfavorable benefit/risk ratio. Indeed, since the probability of developing HAM/TSP or Adult T cell leukemia (ATL) does not exceed a few percent of individuals, lifelong infection with an HTLV-1 strain, even if attenuated, is not devoid of risk. However, lessons from the BLV model are instrumental for the development of immunotherapy against HTLV-1. For example, the BLV vaccine itself could be used as a vector to provide passive immunity. The idea would be to insert sequences encoding HTLV-1 sequences able to induce neutralizing antibodies inside the BLV vaccine backbone. This recombinant virus could be inoculated to lactating cows that would produce milk containing anti-HLTV-1 antibodies. Degenerated codons of B-cell epitopes or mimotopes obtained by phage panning could be used to implement biosafety. The advantage of this approach is the availability of fresh milk in isolated areas where healthcare is limited or unavailable. This is, for example, the case in the high plateaus of northern Argentina (Jujuy) and Bolivia, where HTLV-1 is endemic.

Lessons from the BLV model also emphasize the need for a permanent and efficient stimulation of humoral and cytotoxic immune responses. As indicated by multiple trials and failures, protein-based vaccines are thus very likely not the best option. On the other hand, the most predominant clinical issue would be to stimulate immune response in HAM/TSP or ATL patients. In this context, an alternative immunotherapy for HTLV-1 would involve vaccination against viral factors, such as Tax and/or HTLV-1 bZIP factor (HBZ), using peptides [43] or expression vectors.

## 7. Conclusions

Vaccination appears to be the optimal strategy to reduce BLV prevalence in highly infected regions worldwide. Many previous attempts to obtain a vaccine against BLV were unsuccessful in herd conditions, mainly because of an incomplete or transient stimulation of the host immune response. Currently, the most promising approach is based on the use of an attenuated, but replication-competent, clone that protects against BLV in herds. This vaccine could also potentially be used as a vector backbone to confer protection against other viruses, such as HTLV-1. In the absence of a satisfactory treatment, there is indeed an urgent need for an efficient vaccine against HTLV-1 in endemic regions.
